# Decision making and the bedside assessment: The Speech Language Therapists’ thinking when making a diagnosis at the bed

**DOI:** 10.4102/sajcd.v68i1.790

**Published:** 2021-06-30

**Authors:** Kim Coutts, Mershen Pillay

**Affiliations:** 1 Department of Speech Pathology, University of the Witwatersrand, Johannesburg, South Africa; 2 Department of Speech-Language Pathology, University of KwaZulu-Natal, Durban, South Africa; 3 Speech and Language Therapy, Massey University, Auckland, New Zealand; 4 Department of Health Professions, Manchester Metropolitan University, Manchester, United Kingdom

**Keywords:** speech-language therapy, dysphagia, clinical decision making, clinical swallow evaluation, pulse oximetry and cervical auscultation

## Abstract

**Background:**

The bedside assessment is often seen as a screener because of its high variability in sensitivity and specificity, whilst the instrumental measures are viewed as gold standards because of the ability of speech-language therapist (SLT) to visualise the swallow more objectively.

**Objectives:**

This research article explores how the value needs to be placed on the decision-making abilities of the SLT rather than on the assessment measure itself.

**Method:**

A mixed methodology concurrent triangulation design was employed to collect data in two phases: the first phase included observing seven SLTs conducting assessments using a standardised bedside measure together with pulse oximetry and cervical auscultation. The second phase was a focus group discussion based on the findings from the first phase. Data were analysed thematically using a bottom-up approach.

**Results:**

The following factors were found to influence the decision-making process at the bedside: bedside assessment data sets, patient, multidisciplinary team, context and then SLT. The availability of more data from the assessment from different data sets improved the confidence of the SLT at the bedside when needing to make clinical decisions. Clinical instincts are developed through experience and observation of those more experienced. These skills need to be developed from junior years.

**Conclusion:**

This research study showed that a bedside assessment can provide valuable information that will allow for diagnostic decisions to be made at the bedside. This study also highlighted the importance of critical thinking using clinical instincts, and that these are the factors that need to be valued and emphasised rather than the assessment measures themselves.

## Introduction

Coutts (2019) recently wrote an opinion paper on dysphagia during the coronavirus disease 2019 (COVID-19) era, in which she brought up the topic about the significance around the bedside assessment and the decision-making processes around assessment at the bedside (Coutts, 2019). This research article is a follow-up from the opinion paper and her PhD study.

During the outbreak of the COVID-19 pandemic, the global healthcare systems are under severe strain because of a lack of personal protective equipment (PPE) and a general lack of resources both personnel and otherwise. This resulted in a global limitation of speech-language therapists’ (SLT) access to instrumental measures for dysphagia assessments. This situation, therefore, led to focussing on the decision-making processes and critical thinking around the utilisation of a bedside assessment as the primary assessment measure for diagnostic purposes. This article explores how SLTs make diagnostic decisions when using a bedside assessment and discussing how SLTs use critical thinking in a South African context. This article emphasises the value of critical thinking rather than placing the value solely on the dysphagia measure itself.

### The assessment measures

For the purpose of this study, it is important to make a distinction between screening and assessment. The clinical swallow evaluation (CSE) has typically been more valued as a screening tool as it gathers clinical information from various endpoints (Bours, Speyer, Lemmens, Limburg, & De Wit, 2009). It is also often used as a primer for instrumental measures (Antonios et al., 2010). Videofluoroscopy (VFSS) and Fibreoptic Endoscopic Evaluation of Swallowing (FEES) are considered the gold standard assessment measures because of the detailed visual information that can be obtained (Boaden, Nightingale, Bradbury, Hives, & Georgiou, 2019). The literature and policies from various international bodies have foregrounded the instrumental measures as gold standards. This has then translated into clinical practice patterns. In the South African context, the frequent lack of access to instrumental measures, especially in the public sector where the majority of the population is being treated, opens up the discussion around how SLTs use the information gathered from a CSE to make clinical decisions that is relevant to the context and the patient.

When discussing on decision-making processes, it is important to understand the inter-rater reliability of the different assessment measures. This has been researched before, especially the inter-rater reliability around VFSS.

Despite being a gold standard measure and having the visual confirmation, there is still a high level of disagreement between raters (Scott, Perry, & Bench, 1998; Stoeckli, Huisman, Seifert, & Martin-Harris, 2003). These data show that SLTs can be presented with the same case and reach different clinical conclusions regardless of what measure is being used. This implies that there are other factors that can have an influence on decision-making processes when making diagnostic decisions. Swan, Cordier, Brown and Speyer (2019) took this idea a step further by evaluating the tools used to interpret both VFSS and FEES studies. She concluded that because of the lack of standardisation and statistical significance, there is currently no tool that can be classified as an effective measure to interpret the findings of even these gold standard measures (Swan et al., 2019). Even with visual confirmation, the decisions around what SLTs are viewing appears to be different. This was an interesting finding for the authors of this study as it implies that critical thinking around dysphagia is clearly a complex process that is highly individualised. The findings such as this one start to reveal how clinical decision making and reaching a diagnosis are far more complex than just the assessment measure itself. Perhaps, SLTs need to focus on the clinical data that can be obtained from measures that are available in their context in order to make clinical decisions.

The current challenge with the CSE is that there is significant variability in terms of how SLTs utilise it, both locally and internationally (Andrews & Pillay, 2017; Bateman, Leslie, & Drinnan, 2007; Rumbach, Coombes, & Doeltgen, 2018). This variability, in practice, often revolves around what aspects should be included in a CSE protocol. What is well known is that in isolation the CSE has a highly variable accuracy (Lynch et al., 2017). The other aspects, such as cervical auscultation (CA) and pulse oximetry (PO), when used in isolation also have poor reliability (Britton et al., 2018). However, when looking at including measures such as CA with the CSE, reliability of both measures can be improved. A study combined CA with a water swallow test to determine the presence of dysphagia (Watanabe et al., 2020), which revealed that even non-swallowing experts were able to accurately detect dysphagia using these methods. Bergström, Svensson and Hartelius et al. (2014) carried out an interesting study evaluating the use of CA together with the bedside assessment and then compared their findings with FEES. Their findings revealed that if an SLT is trained in CA, then by adding it as an adjunct to the CSE, can improve its sensitivity to detect the presence of dysphagia, and this can be more accurate than just the CSE in isolation (Bergström et al., 2014). This was supported by another study, which suggested that CA can be used as an adjunct to the bedside assessment (Frakking et al., 2016), the findings of which were also supported in a systematic review carried out by Bours et al. (2009). This an important study that evaluated all of the current literature in the field regarding dysphagia bedside assessments at the time, the conclusions of which showed that there is a significant value in using multiple data sources at the bedside, such as the CSE and the CA.

In terms of pulse oximetry, a systematic review by Britton et al. (2018) indicated that because of the lack of standardised protocols and interpretation of findings, it too should not be used in isolation to detect the presence of dysphagia but some studies have concluded that PO can be a helpful adjunct to the bedside assessment as it provided relevant physiological data (Britton et al., 2018; Colodny, 2000; Zhou, Salle, Daviet, Stuit, & Nguyen, 2011). At the bedside, the use of each of these measures in isolation is clearly not recommended but when using them simultaneously is a helpful adjunct. This is because there are more clinical data on which SLTs can make a clinical decision.

There is a significant clinical value in all assessment measures. However, when looking at policies, and the research, instrumental measures are often foregrounded. Given the high variability around decision making in all of the assessment measures, there is a need to shift what is valued in the assessment process. Dysphagia assessments are not solely about the use of a particular measure but that the value is rather in the critical thinking of the SLT pertaining to how the available clinical data from these measures are being used (Plowman & Humbert, 2018). Given the complexities of COVID-19 era, this article has chosen to focus on the critical thinking around the CSE as SLTs will need to be making diagnostic decisions at the bedside more frequently, and thus, this process requires more exploration, especially in the South African context.

### Clinical decision making

The South African healthcare context is complex when compared with other developed countries. The contextual limitations of access to skilled staff members, instrumentation and health profile mean that SLTs often have to make clinical decisions based on multiple contextual, patient, linguistic and cultural factors. How this is carried out has not yet been explored.

Speech-language therapists have started to research on the impact of critical thinking and decision-making processes in dysphagia; however, these studies are based in economically developed countries. Although findings from developed countries are useful, it may not be easily extrapolated to fully understand what happens with SLTs in South Africa. A study by Tohara, Saitoh, Mays, Kuhlemeier and Palmer (2003) made an interesting suggestion when evaluating the inter-rater reliability of FEES, suggesting that when an evaluation criterion, that is, a standardised form, is available, the decision-making process improves (Tohara et al., 2003). The same study also mentioned the importance of clinical experience in this decision-making process. This finding was linked to Vose, Kesneck, Sunday, Plowman and Humbert (2018) who evaluated the clinical decision making of SLTs when evaluating a VFSS. They concluded that there was significant variability amongst SLTs, and that the key contributing factor was the lack of consensus and understanding of what a normal swallow was and what are normal deviations (Vose et al., 2018). These findings were linked to an Australian study that assessed the variability of practice patterns for the bedside assessment and what implications this had for SLTs (Mcallister, Kruger, Doeltgen, & Tyler-Boltrek, 2016). Their findings suggested that rather attempting to find standardised items on a recording form that rather the clinical value needs to be placed on the decision-making process itself and what information SLTs used to make conclusions.

These findings seem to imply that clinical decision making is a multifaceted process that is influenced by various factors. Humbert (2015) suggested that decisions are made by integrating evidence together with experience, which develops clinical instincts that is developed over time (Croskerry, 2009). The study by Pillay and Pillay (2020) developed a dysphagia clinical reasoning framework that echoes the findings from Humbert (2015). This framework suggests that junior SLTs move along a novice to expert continuum by gaining clinical knowledge and experience together with understanding diseases and their progression. At the end of the continuum, through the combination of the different factors, these SLTs develop clinical reasoning, which can be used in making decisions at the bedside when assessing dysphagia.

Considering the factors above, the aim of this study was to describe the decision-making processes of SLTs when conducting a CSE. This research study stemmed from a doctoral study that focused on developing an integrated bedside assessment method. The focus of this study is on the data obtained from the decision-making processes of the SLTs when using these measures. In an everchanging world, given the current dysphagia guidelines in place (SASHLA, 2020), this shift in how SLTs use information from a CSE to make a diagnosis is becoming increasingly important, especially in the complex SA healthcare context. The research question was how do SLTs make the decision regarding the presence of dysphagia, aspiration or penetration at the bedside?

## Study objectives

This study used a mixed methodology concurrent triangulation design to address the following objectives:

- Describe what factors SLTs use to determine the possible presence of dysphagia, penetration and/or aspiration at the bedside.
- Explore how the SLTs used these factors to make these decisions at the bedside.

### Site

This study was conducted at a tertiary-level public sector hospital in Johannesburg. The public sector was chosen for this study as it serves over 78% of the population with minimal resources, both personnel and otherwise (Coovadia, Jewkes, Barron, Sanders, & Mcintyre, 2009).

### Participants and sampling method

Convenience sampling was used to obtain a sample group of seven SLTs who worked at the research site presenting with different levels of experience from 1 to 8 years. All participants included were female. The two participants who had the most years of experience had been colleagues for 4 years, and three were registered as SLTs and audiologists. The four participants with the least experience were registered as SLTs.

### Data collection tools

The Mann Assessment of Swallowing Ability (MASA) (Mann, 2021) was used as the CSE protocol for this study. It is a standardised tool that assesses the anatomical and physiological aspects of the patient’s swallow, with different swallowing trials being conducted at the bedside. It was standardised on an adult neurogenic population. This tool has proven validity and reliability for clinical use in a variety of adult populations (Chojin et al., 2017; Ohira et al., 2017). At the end of an assessment, the SLT participants classified the outcome on a ranked scale as follows: 0 – no abnormalities detected (NAD), 1 – dysphagia, 2 – penetration or 3 – aspiration for analysis purposes. For the purposes of this study, the ranking of a score of *1* = dysphagia (oral and pharyngeal), which was defined as difficulty with swallowing but no presence of penetration or aspiration. The ranking of a score of 2 = penetration, which was more severe than 1 as it represents the presence of an oral and/or pharyngeal dysphagia and penetration. A score of 3 was the most severe and was defined as the presence of aspiration. The CA aspect allowed the SLTs to classify the swallow sounds at the start, during and end of the swallow. The PO readings were collected prior to the assessment as a baseline, during and 2 min post swallow. The same ranked outcomes of 1–3 were used for PO and CA. The researcher used an observation schedule to conduct the bedside observations. This schedule included aspects around SLT, patient, environmental or contextual and multidisciplinary team (MDT) factors.

The data collection schedule for the focus group (FG) discussion included a semi-structured questionnaire that focused on asking key questions on the decision-making processes based on the clinical factors indicated by the SLT for each case, as well as the observation notes. The researcher would allow the SLT to explain how they reached the conclusion of dysphagia or aspiration or penetration based on the clinical information that was recorded at the bedside. Clarification of concepts was made during the interview process.

### Data collection

#### The first phase of data collection

Patients with a neurogenic dysphagia were assessed within 24 h using the MASA and the CA and PO procedures as set out by Cichero (2006). Each patient was then re-assessed 24 h later by a different SLT using the same procedures. This was a blinded process. Simultaneously, the researcher conducted observations for the different SLTs using an observation schedule to understand the bedside assessment environment and to enhance the discussion in the FG.

#### The second phase of data collection

After analysing the results from the bedside assessments and observations, the researcher looked for any differences in outcomes between the two SLT participants for the same patient. These different outcomes, as well as the notes from the observations, were then discussed in a FG with all the participants.

### Data analysis

The data from the FG discussion were analysed thematically using a bottom-up approach (Braun & Clark, 2006). The transcriptions were analysed using the four phase analysis process suggested by Vaismoradi, Jones, Turunen and Snelgrove (2016), initialising of the data, construction of themes, rectification of themes and finalisation of the story line.

#### Trustworthiness

The use of triangulation method, that is, using more than one data collection method improved the credibility and transferability of the findings (Heale & Forbes, 2013). The three different data sets included the CSE assessments, the observations and the FG discussions.

### Ethical considerations

Ethical approval was obtained from the University of KwaZulu-Natal Biomedical Research Ethics Committee, reference number: BE362/15. Participant consent was obtained prior to data collection.

## Results and discussion

### Objective 1: Describe what factors speech-language therapists use to determine the possible presence of dysphagia, penetration and/or aspiration at the bedside

**FIGURE 1:**
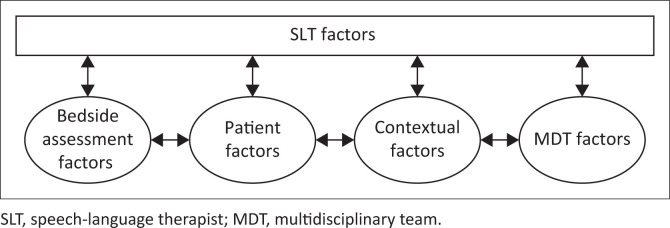
Decision-making factors when using a bedside assessment.

Based on the data obtained from the first phase of data collection, the following themes were identified: the factors from the bedside assessment, the patient, the multidisciplinary team, the context and then the SLT factors. The relationship between these factors is depicted in figure 1.

There are different factors that inform the overall decision, but SLT factors are used to integrate the information and make the overarching clinical decision. How these factors work together and allow for integration will be explained under objective 2.

### Objective 2: Explore how the speech-language therapists used these factors to make these decisions at the bedside

‘…as the oxygen saturation levels did not drop and I did not hear any gurgling, delay or bubbling…’ This statement made by SLT 4 in the FG discussion depicts the integration needed in the decision-making process quite aptly. It shows how an SLT uses physiological data from different assessment measures to make a diagnostic decision at the bedside. This statement will be deconstructed in the discussion according to the different factors that were identified above.

### The bedside assessment factors

All participants were observed to have followed a similar core CSE protocol, which included the case history, oral sensory motor examination, language and speech screening followed by food trials, usually starting with a liquid. This stems from their understanding of the theoretical knowledge relating to dysphagia assessments. The general status of the patient was always considered prior to the start of the assessment, which included the level of alertness or cognitive functioning, factors such as oxygen support and patient compliancy.

On average, the bedside assessment that included PO and CA evaluations provided more physiological data when compared with the use of the CSE alone. When using the CSE alone, the SLTs used one to two data sets on which to make a clinical decision at the bedside as to whether the patient presented with just oral dysphagia or possible penetration and/or aspiration. When using the CSE with PO and CA, the SLTs used three to four data sets. This extra data improved the SLT confidence, as seen in the opening statement from SLT 4. This is also supported by SLT 6 who stated that ‘she … felt more confident with CA’.

#### The presence of aspiration

When deciding on the presence of aspiration using the MASA, all SLTs commented on the presence of decreased laryngeal elevation followed by a gurgly vocal quality or wet sounding chest and poor cough. For the CSE with PO and CA, a diagnosis of aspiration was seen using aspects, such as multiple swallows, decreased breathing abilities, a drop of more than 3% on oxygen saturation levels and a gurgly vocal quality. Together with the patient observations, the data from PO and CA allowed the SLT participant to either further suspect aspiration or penetration. This was confirmed by SLT 4 as mentioned in the opening statement. Conversely, SLT 3 cited about another patient in the FG discussion, line 153, ‘she showed significant signs of aspiration with coughing, and choking and I heard a change in breathing with an 11% drop in oxygen saturation’.

#### The presence of penetration

A diagnosis of penetration was rarely made and only seen once in the patients where there was a difference in outcomes between two SLTs. The diagnosis of penetration was made using the bedside assessment with PO and CA, and the SLT used poor tongue movement, gurgly vocal quality, wheezing and multiple swallows as key indicators. An interesting finding was that this diagnosis was also made by the most experienced SLT participant in the department. The more experienced SLTs tended to not diagnose the patient with aspiration but rather dysphagia or penetration when compared with the more inexperienced SLTs in the department. As the symptoms of penetration are more subtle, perhaps clinical experience leads to improved confidence when deciding on the more subtle presence of penetration. This theme of experience will be discussed more under SLT factors.

#### The presence of dysphagia

In the case of the diagnosis of dysphagia, the most common diagnosis made by all participants, the clinical symptoms that were used were residue in the oral cavity, oral weakness as seen from the oral sensory motor examination or the presence of a motor speech disorder, poor bolus control and delayed oral transit time. This was possibly the more common diagnosis as these are the symptoms that are more easily identifiable at the bedside.

In summary, in terms of the bedside assessment protocol that she used, SLT 5 stated that:

[*I*]n community service, you follow a strict protocol because that is what you know and feel comfortable with, with experience and being exposed to different patients, you then change the protocol as you see fit. (p. 5)

This was an interesting statement that was linked to the concept of confidence that can improve critical thinking. It became apparent that SLTs with more experience were willing to make a diagnosis of penetration and/or aspiration more easily than those with less experience. When the SLTs were diagnosing penetration and/or aspiration, the availability of the data from CA and PO further assisted them in the decision-making process rather than just the data from the bedside assessment. As aspiration and/or penetration cannot be seen from the bedside, SLTs valued the availability of the extra physiological data.

### The patient factors

There is a need for the bedside assessment protocol to be adapted to accommodate the requirements of the patient. Patient compliance and language were significant sub-themes that emerged in observations, SLT 1 stated that ‘patient factors can definitely affect my ability to conduct a good assessment at the bedside,’ and SLT 4 confirmed this by referring to ‘the use of instrumental measures in an assessment protocol that is highly patient dependent’ and with SLT 6 stating ‘…it all depends on the patient’. This is a pertinent challenge when working with patients who have neurological conditions, as well as in a public sector hospital where access to instrumental measures is a further challenge. The need to use instrumental measures and when to refer a patient for one was closely related to SLT knowledge and experience. The patient compliance and language abilities will also influence the assessment findings in terms of the information that the SLT can obtain from the patient and how accurate their assessment may be. This can be highly pertinent for patients with neurological impairments, such as CVA - cerebrovacular accident and TBI = traumatic brain injury (Zimmerman, Murdock, & Shank, 2010).

### The multidisciplinary team factors

The frustration of SLT participants became apparent with the challenges that can arise from working with other professionals when assessing dysphagia at the bedside. SLTs 1, 2 and 4 expressed that their greatest challenge was working with the nursing staff. SLT 1 noted that ‘the input that you receive from the nurses is often inaccurate in terms of whether they are tolerating their current feeds’. SLT 2 mentioned, ‘often the information that is on the balfec (intake) chart is incorrect’. SLT 4 further supported this by saying ‘I take the opinions of certain staff members with a pinch of salt’. This led the participants to consensus in the FG of needing to rely on their findings in the bedside assessment, often above what the other professionals had described.

In opposition to that, the discussion with other professionals (including nurses) was important for some participants as mentioned by SLT 2 who stated that ‘I chatted to the nurses about her eating…’ as she wanted to find out how the patients were presenting during other mealtimes. SLT 7 mentioned, ‘… I like to see what others have said…’ This was to acquire a holistic picture of the patient. An SLT also had a discussion with the family during one of the assessments. There were discussions with other members of the team, which contributed to decision making by the SLTs as this provides input from different situations. SLT 4 was also able to observe the occupational therapist feeding her patient, which allowed for a different view on the eating process as said by ‘you need to take other professionals point of view into account…’

### The contextual factors

The challenge of a high patient load was also pertinent, and the participants made note of the importance of time and resource efficiency for the assessments. Some assessed two areas whilst performing one activity in order to save time, that is, receptive language simultaneously with an oral sensory motor examination. Another efficient method was using food that was readily and easily available, and replicated a functional eating situation. SLT 1 mentioned that ‘I need to make a plan by creating an easy ward kit which helps to conduct full assessments at the bedside’. SLT 4, who had more clinical experience, said, ‘I can conduct a good assessment and not have anything on me but purely with what I find in the ward’. Whilst their ideas were the same, their approaches were different. The differences in their approach could be because of experience in the field, as well as the contextual factors. The participants would often try to use food that was readily available at the patient’s bed or what came from the ward in order to achieve an adequate assessment at the bedside, as this also helped to assess the patient in a more natural eating situation. It needs to be noted here that this could be a distinct advantage of using a bedside assessment over an instrumental measure, the need to assess in a realistic environment, otherwise known as ecological validity (Andrade, 2018). One of the more senior SLTs summed up this discussion on resources well in relation to having limited access to FEES that …‘I feel that we are doing excellent assessments without barium swallows’. This was an important statement as she felt that adequate clinical decisions were being made at the bedside by SLTs.

### The speech-language therapist factors: How it is all pulled together

The decision-making process at the bedside is strongly associated with SLT knowledge and experience in dysphagia. The junior SLTs were more inclined to talk about clinical factors that related directly to procedural knowledge as found in statements, such as ‘I decided to start with water as it is the safest consistency…’ (SLT 3, Line 40) and ‘I needed to position the patient appropriately first…’. SLTs 2 and 3 both referred to using three trials of one consistency before making a decision. Another junior SLT highlighted that ‘[*i*]t is a confidence thing that comes from experience’. These quotes highlight that as junior SLTs have less experience that they felt the need to actively mention procedural or theoretical facts that perhaps more experienced SLTs just do instinctually.

What is a novel finding, this research observed a strong theme of clinical instincts, and it was clearly evident that the SLTs gained experience through practice. This was described in ways such as ‘practice makes perfect’ (SLT 2) and ‘I make decisions based on outcomes from previous cases’ (SLT 1). Interestingly, three of the participants mentioned the use of ‘gut feelings’ or ‘instincts’. SLTs 2 and 5 also made a statement to support this, ‘…I also know from my previous experience…’ and ‘[*i*]t is a confidence thing that comes from experience’. SLT 2, with 1 year of experience, and SLT 3, with 3 years, both mentioned that they were ‘learning to trust my gut a bit more’ when making a diagnosis at the bedside but the extra physiological data did assist in this process. It appeared that the more experience SLTs had, the more inclined they were to trust the results of the bedside assessment and their clinical instincts. This was confirmed by SLTs 1 and 2 who also mentioned *instincts* and ‘trusting their gut’. This concept of experience better assisting in decision making is supported by the finding that the senior SLT was the only participant who was comfortable to diagnose the more subtle ‘penetration’ instead of choosing ‘aspiration’ as an outcome. These findings link clearly to the dual processing theory, as suggested originally by Croskerry (2009) and then later by Humbert (2015). This notion of experience influencing decision making was also referred to by Pillay and Pillay (2020, in press).

It is important to note that some people learn in different ways as SLT 3 seemed to rely on external sources for her decision making rather than purely on her clinical instincts. This was evident in the comments, ‘I like to discuss the case with the doctor first’ and ‘I gained my experience from drawing on the knowledge of others and reading. I also found that attending courses was helpful for me’. This raised an interesting point as the relationship between colleagues may then have a role to play in this learning and/or mentoring process. This highlights the importance of placing junior SLTs in facilities where they can be mentored by more experienced SLTs in dysphagia, as this is a valuable learning opportunity (Coutts, 2019).

In summary, there were a variety of terms used by the participants throughout the FG discussion, which signified what cognitive factors SLTs use to make decisions at the bedside. These factors around critical thinking included aspects around praxis defined as the interdependence and integration of theory, practice, research and development, thought and action (Whitehead, 2002). Praxis is a completely individual process and proves that no assessment measure is without some element of subjectivity. This links to how participants used other factors, such as experience and gut feelings, to make these decisions. Table 1 summarises these cognitive factors and the frequency of their occurrence:

**TABLE 1:** Cognitive factors used and the frequency of occurrence.

Terms used	Frequency of occurrence
‘Weary’/‘worried’/‘I felt (un)comfortable about…’	13
‘…From my experience…’	5
‘…from my suspicions…’	2
…my gut feeling…’	3
‘I felt confused…’	2
‘… I wanted to chat about or discuss with…’	2

It was apparent that the SLTs gained knowledge and experience in different ways, which resulted in diverse ways of developing clinical thinking or as this study could suggest, clinical instincts. One of the participants summed it up well and supports the use of the term ‘clinical instincts’ when she said, ‘It made me realise how much of my decision-making is unspoken and based on very subtle things … it was difficult to explain those subtleties…’. This is important when considering undergraduate teaching, as well as mentorship for junior SLTs as they proceed into their community service year (Coutts, 2019). As shown in the table, clinical instincts are a predominant feature of critical thinking, and needs to be nurtured and developed from junior years.

## Implications

### Motivation for a bedside assessment measure

This research study has started to explore the potential value of a bedside assessment through the decision-making process when extra physiological data are available. This study further confirms findings from previous studies, which suggest that when using a CSE with CA and PO, it improves the decision-making process at the bedside. This is because of two aspects, the first is by Bours et al. (2009) whose systematic review highlighted the value of an integrated bedside assessment using different measures because the use of extra measures improves the overall reliability.

The second is that by having the extra physiological data, there is increased confidence to make decisions at the bedside. The ability to make decisions at the bedside not only assists with making a diagnosis but is also important for future therapy planning. Most participants regardless of their experience followed a similar bedside assessment protocol; however, this process needs to be flexible as it can be influenced by patient, MDT and contextual factors. The ability of an SLT to make appropriate changes requires critical-thinking skills, which are developed through experience and from the observation of more experienced SLTs in the field.

### For clinical decision making

Clinical decision making has been studied in a variety of other healthcare professions, including physiotherapy, nursing and medicine. The physiotherapy studies have assisted in developing student training and shaping education (Smith, Higgs, & Ellis, 2008). The nursing studies have resulted in the development of models, which also has an impact on student training as well as guidelines (Hoffman, Donoghue, & Duffield, 2004). The area of decision making is relatively new in the field of dysphagia and has not yet been investigated in the South African context. This study has, therefore, provided theoretically novel data as the starting point for future research. This study has also provided data on how decision making occurs during a CSE; data from this perspective are also novel in the field.

The findings from this study echo those from Croskerry (2009), Humbert (2015) and Pillay and Pillay (2021, in press). Decision making is a complex process influenced by various factors but the key component to this is how clinical instincts and confidence to make diagnostic decisions are linked to clinical experience. For the SA context, this is helpful in terms of needing to potentially revise undergraduate training and looking at how community service is potentially structured. Given the complexities of the SA healthcare system and the recent shift to online teaching pedagogies, it is imperative to understand how to foster and develop these critical-thinking skills in students along with the development of their clinical skills. By understanding how junior SLTs move along this continuum can assist academics and those involved in student training.

By harnessing the reliability of an integrated bedside assessment and improving the integration of the various patient and contextual factors can then advance the overall delivery of an efficient, real time, contextually relevant dysphagia service.

These data were gathered before the COVID-19 pandemic, although relevant, the data need to be potentially updated as the patient, MDT and contextual factors will need to include the factors that COVID-19 brings with it, such as social distancing.

## Conclusion

The science of dysphagia, our research methods used in dysphagia and how SLTs align those into research papers should become more integrated to what occurs in the real world. Pillay, Kathard and Samuel (1997) argued for a shift in how we use our science to practice in the real-world context. They referred to science as knowledge that was constructed from and of the real-world context. This study further confirms that a positivistic science is unsuitable for engaging dysphagia practitioners in clinical thinking. Unlike a positivist (or empirical) science that seeks to establish a singular truth via the myth of objectivity, we argue that dysphagia practitioners need a more critically oriented science. Such a science should celebrate, not deny, subjectivities, such as that articulated by the participants in this study. This may better facilitate thinking, holistic and ecological system-based approaches as useful paradigms for bedside or CSEs. This article suggests theoretical novelty in how clinical decision making is carried out within a healthcare setting by SLTs with varying levels of experience. This is a complex process that requires multiple levels of analysis; however, the hope is that this study has started to contribute towards how SLTs in South Africa move along this novice to expert continuum. This study suggests that the priority needs to be placed on the development of the critical-thinking processes rather than on the measure itself when developing clinical competence. This needs to be honed from undergraduate years. Critical thinking is based on developing clinical instincts, especially when making decisions at the bedside. These skills are developed predominantly through clinical experience and observation of others. These data can possibly assist in future training of both undergraduate and postgraduate students.
